# Regional Differences in Chinese Female Demand for Childcare Services of 0–3 Years: The Moderating and Mediating Effects of Family Childcare Context

**DOI:** 10.3390/children10010151

**Published:** 2023-01-12

**Authors:** Chuanfang Chen, Huimin Hu, Renbing Shi

**Affiliations:** School of Sociology, Huazhong University of Science and Technology, Wuhan 430074, China

**Keywords:** demand for childcare services, regional differences, family childcare context, moderating effect, mediating effect

## Abstract

There are multiple reasons to consider the use of formal childcare: parental employment, child development, fertility choices, elderly health, generational relations, etc. This study explores the relationship between regional differences (urban/rural; eastern/central/western) and demand for childcare services (quantity, price, quality) from birth to three years of age, moderated and mediated by the family childcare contexts among Chinese women. Altogether, 1770 mothers of children aged 0–3 were selected from a national survey and analyzed. There are three major findings: (1) Urban mothers show a willingness to spend on the higher monetary cost of center-based childcare compared to rural mothers, as a result of more severe work–child conflicts faced by urban women. Urban–rural gaps in individual and household income also contribute to the differences in affordability. (2) Mothers in eastern China have a more substantial need to place their infants or toddlers in nurseries before the age of three than their counterparts in central and western China, primarily due to a lack of grandparental and paternal childcare support and an expectation of higher quality programs. (3) There is no significant regional disparity in terms of care-related or education-related quality preferences. The paper proposes regional prioritized strategies and targeted services to address the “3A” problems of childcare provision.

## 1. Introduction

Problems in the provision of childcare services at birth to 3 years of age in China are termed the “3A” problems: accessibility (difficulty in obtaining childcare services), affordability (paying expensive fees), and accountability (poor quality and little supervision) [1,2,3]. These “3A” problems keep families and women from easily using childcare services, which is detrimental to increasing birthrates [4,5]. To tackle these challenges, the Chinese central government has introduced a series of policies to promote the development of childcare services for children before preschool since 2019, e.g., Guidance on Promoting the Development of Care Services for Infants under 3 Years Old, issued in 2019 [6]. However, despite government efforts in recent years to make high-quality care and education services available at lower prices for more infants and toddlers, the “3A” problems still cannot be alleviated sustainably and equally in certain regions [7]. Accessible, affordable, and high-quality childcare services are scarce, necessitating sustained and increasing investment of public funds. Therefore, the supply of childcare services for 0 to 3 years in China is facing a difficult dilemma: at least one target of “3A” cannot be achieved, or “3A” can only be satisfied in the short term or unequally among eligible children.

Researching the characteristics of the demand is fundamental to solving the supply dilemma and meeting childcare needs effectively. It is well-known that China’s economic, cultural, family, and educational developments, namely, sociocultural contexts, have significant differences among various geographical locations, and these regional disparities could affect childcare services [8,9,10]. As Hong et al.’s [2] study mentioned, future research should compare childcare services considering geography locational and urban–rural disparities to better understand internal heterogeneity in China. Regarding urban–rural differences, the need for infant–toddler services among urban parents is generally more urgent and higher than that of rural parents [11,12]. A few studies predict that although the population of children requiring preschool education in rural areas will decline in the future, the shortage of preschool education resources in rural areas is still a significant issue compared to in urban areas, due to huge gaps in economic development and financial investment [13,14,15,16]. For eastern and central/western differences in demand for childcare services, most existing studies have briefly analyzed or mentioned this inter-regional imbalance in the development and demand of childcare services, but only within a city or province rather than the whole country [7,17]. Moreover, Hong et al. found that parents from different locations have varying degrees of satisfaction with early childhood education services [10]. Nevertheless, the literature on regional differences in demand for childcare services for under three of age is limited, and few existing studies are comprehensive and systematic.

The paper will focus on Chinese mothers rather than parents, based on structural and cultural assumptions about parenting responsibilities. In a Chinese sociocultural context, mothers and grandparents take on most of the responsibility for children’s care. CFPS data show that 45.8% of children aged 0 to 5 years are cared for by their mothers during the day, which increases to 66.7% at night. Only 1.7% and 3.7% of fathers are the primary caregivers of infants and toddlers during the day and night, respectively [18]. Mothers usually face more severe work–parenting conflicts than fathers. Therefore, focusing on and addressing maternal childcare needs is more necessary. In addition, there are multiple reasons to consider using formal childcare in China: parental employment, child development, fertility choices, elderly health, generational relations, etc. Indeed, family childcare arrangements are embedded in a complex and dynamic context involving culture, beliefs about care, personal experiences, parental needs, information, and resource constraints [19,20]. Two kinds of recent theoretical and empirical studies in the literature explain how this context functions. On the one hand, economic studies have generally analyzed childcare as parents’ labor supply and consumption choices, assuming that these choices reflect parenting preferences and budget constraints. On the other hand, other social scientific research has examined the nature of choice, which relates to social structures and processes that further influence parental beliefs of care, information about options, and resource constraints [19]. In Western countries, non-parental childcare arrangements serve two main purposes: to help parents participate in the workforce and promote children’s growth and development [20].

There are substantial regional differences and unique contexts in China’s economy, society, culture, family, and education, which could spill over into childcare expectations. Therefore, investigating the regional differences in demand for childcare services combined with the unique Chinese sociocultural context is necessary, especially given that a limited amount of literature has contributed to this field. This study aims to examine the association between regional differences (urban and rural areas; eastern, central, and western China) and women’s diverse demands (quantity, price, quality) for infant/toddler care services, moderated and mediated by the family childcare setting. To conduct this research, we will use secondary data from the National Population and Household Dynamics Monitoring Survey in 2019. Taking into account the pressing issue of implementing the Three Child Policy and actively coping with the aging of population in China, the findings will provide reference and inspiration for policymaking and practice improvement of early childcare provision.

## 2. Literature Review

### 2.1. The Dilemma of China’s Childcare Service Provision

This study will mainly focus on the childcare services provided to children aged 0 to 3, which have been heavily neglected by government and social services ever since the 1990s. Preschool programs in China are mainly composed of two levels: the first is the childcare program provided by childcare institutions for infants or toddlers under 3, and the other is the education and care services offered by kindergartens for children aged 3 to 6 years [7]. Compared with the boom in early education participation (3–6) provided by kindergartens since 2010, infant/toddler care services (0–3) remain an underdeveloped area [21] in need of further scientific research and policy intervention. Currently, two types of institutions provide childcare services for young children under 3 in China: center-based nurseries and early learning centers. The nurseries are similar to daycare centers in other countries, which specialize in offering professional custodial services, with some centers also providing boarding facilities. Early learning centers are emerging as an option and are very popular among Chinese parents. They aim to develop children’s potential motor, language, interaction, and intelligence skills and assist other childminders in improving their caring or parenting skills in parent–child classes [21]. This paper applies the concepts of supply and demand in economics to childcare services, defined as follows. The demand for childcare services refers to the desire to obtain care and education services and the corresponding ability to pay for them. On the other hand, the supply of childcare services refers to the custodial and educational opportunities and resources that the administrators and providers are willing and able to provide. Both demand and supply are related to three dimensions: quantity, price, and quality [22].

China’s childcare service development is at a critical turning point, with numerous obstacles. From a historical perspective, since the founding of the People’s Republic of China in 1949, care services for infants and toddlers from birth through to 3 years of age have experienced a meandering development process accompanied by political and economic transformations [23]. The provision status of childcare services for 0 to 3 years in China is far from accessible in quantity, affordable in price, and accountable in quality (i.e., the “3A” problems), evaluated through a compelling “3A” framework proposed by Li et al. [24]. First, there are not enough services available. In 2017, the enrollment rate of infants and toddlers under three reached 5.5% in mainland China; in contrast, up to 68.4% of families have childcare needs [25]. Moreover, there is also a large gap compared with developed countries. The average enrolment rate of children aged 0 to 2 years in OECD countries reached 30%, particularly in Denmark, Belgium, Iceland, France, Israel, the Netherlands, and Norway. Japan, South Korea, and Singapore, which share East Asian cultural aspects with China, also placed more than 20% of infants in childcare institutions [26]. Second, the price of services is not affordable. More than half of families reported their expenditure on childcare was too high, which accounted for 12% of total household income [25]. Most childcare services for children under three are supplied by private for-profit institutions rather than public welfare institutions. The statistics show that 26 million children aged 0 to 3 years old enrolled in various childcare institutions in 2020, including private institutions (49%) and education departments (43%) [27]. Third, the quality of services provided fluctuates across centers. Many existing institutions and teachers are underqualified and do not fall under the supervision of public administration. In addition, daily care, and education, both of which are equally critical to healthy infant and toddler development, are not delivered equally in some childcare settings. Consequently, the scarcity of high-quality childcare has become an obstacle for parents trying to find a nursery. Most parents are hesitant to send their children to nurseries without guaranteed service quality [28,29,30].

However, previous studies addressing the dilemma of childcare provision have yet to pay attention to compatibility or priorities among policy objectives. To solve the “3A” problems, research scholars have put forward plenty of countermeasures to increase supply quantity, lower service prices, and boost service quality [31,32,33]. Even so, sufficient quantity, low cost, and suitable quality of childcare services require continuous high fiscal budgets (which cannot always be satisfied by local government). Consequently, the three goals of 0–3 childcare services are hard to realize simultaneously [7,34]. In other words, at least one policy objective cannot be achieved, or all three goals can only be maintained in the short term instead of the long term or with a limited number of children. This study aims to provide recommendations from a demand-side point of view.

### 2.2. Demand for Childcare Services and Regional Differences

There have been numerous studies on demand for childcare services (DCS) for children aged 0 to 3 years, which have mainly focused on two parts: one is to analyze the preferences or characteristics of childcare needs, and the other is to explore the influencing factors of the needs. In the existing domestic literature, most studies explore the preferences and characteristics of Chinese residential demand, mainly based on specific survey data of urban areas. Li et al. [11] found that parents in Nanjing prefer childcare institutions with good outdoor activity areas, professional and affectionate teachers, and surveillance cameras. Hong et al.’s [35] research, based on 12 cities, revealed that young families show dissatisfaction with childcare services. Families with children aged 2 to 3 have the most urgent needs for full-day care. The needs for early education are higher than those for daily maintenance. Shi and Liu’s [12,36] work showed a hierarchical and diversified trend in childcare needs, as parental needs and choices for institutional infant/toddler services are influenced by complex factors, both inside and outside the family. Huang’s [37] research in Changsha points out that nurseries have a large student pool with its newborn population and great potential from the perspective of the macro market; in contrast, it has high requirements but low trust from the micro perspective of the people. Gao et al. [38] found that childcare services are at the first level of policy needs for people of childbearing age, indicating the demand preferences of “safe dominantly, integrating care and education, affordable”. Urban parents are most willing to pay for monitoring equipment, and willingness to pay for other facilities decreases in sequence. In the study by Dan and Jiao [39], parents attach great importance to the quality of childcare services, with qualified teachers, health care, and an appropriate environment as the leading factors in Shenyang. However, Shi [40] concluded that the needs of Chinese families for childcare services are generally urgent but lack comprehensive and in-depth research.

Regarding prior studies performed outside of China, scholars focus on exploring family- and institution-related influencing factors of DCS. First, some of the literature has examined the impact of family factors such as parent ethnicity/race, income level, education attainment, employment status, and the number of children on DCS. Immigrant families, such as those from Mexico, Asia, and Spain, are less likely to send their young children to nurseries and prefer household childcare to care outside of the household [41]. Infants and toddlers from high-income families are more likely to participate in early childhood education and care programs than those from low-income families [42]. Parents whose school attainment and personal beliefs are consistent with those in childcare centers are most likely to use program subsidies and place their children in centers [43]. Full-time working parents tend to place their children in a nursery to alleviate severe work–family conflict. Mothers with higher income and shorter working hours are more likely to base their choices on quality rather than practical concerns. Mothers with increased parenting stress are more likely to consider practical concerns [44,45]. Age and number of children may be related to childcare choices [46]. Second, others have explored the influence of institutional factors, such as location, cost, and quality, on DCS. Johansen et al. [47] divided the institutional attributes into intrinsic features, such as appropriate toys, curriculum, and group size, and extrinsic features, such as location, cost, and operation hours. Intrinsic factors are widely considered by parents when they decide on childcare, as previous studies have shown [48]. Location convenience is crucial for dual-working parents when choosing a care facility for infants and toddlers [49]. While parents are acutely sensitive to the cost of childcare, they also strongly prefer quality, particularly in terms of the educational attainment of the caregivers [50].

### 2.3. Family Childcare Context and Facing Challenges

**Figure 1:**
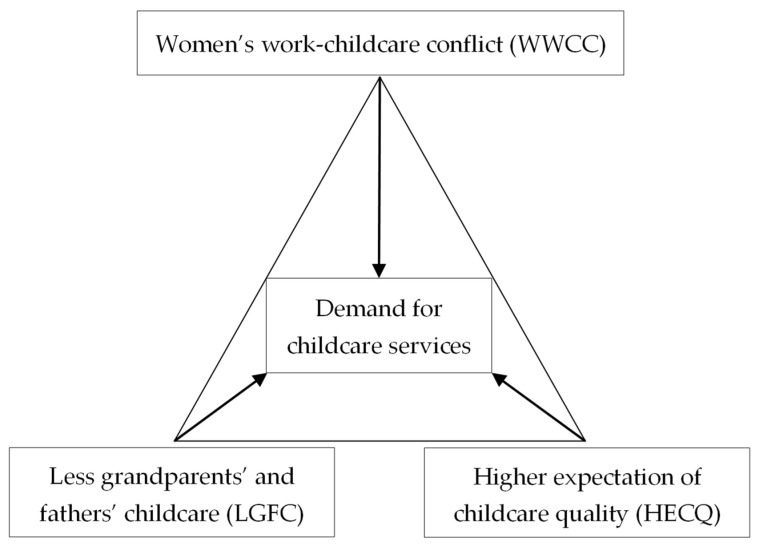
The Chinese context of family childcare for children under three.

In the Chinese context, the stakeholders of family childcare (informal care) and non-family childcare (formal care) arrangements primarily include mothers, fathers, grandparents, and very young children. Through referring to relevant research [2,20,21,22,23,24,25,26,27,28,29,30,31,32,33,34,35,36,37,38,39,40,41,42,43,44,45,46,47,48,49,50,51], the family childcare context (FCC) of mothers’ demands for childcare includes three categories in China: women’s work–childcare conflict (WWCC), less grandparents’ and fathers’ childcare (LGFC), and higher expectation of childcare quality (HECQ), as shown in Figure 1.

Firstly, women’s work–childcare conflict (WWCC) has become more intense. Chinese women’s labor participation rate is markedly higher than that of most countries in the long term, and young mothers have to bear a “dual burden” as both caregivers and earners [52,53]. Although China has extended maternity and parental leave in recent years, many studies have shown that the longer women leave the labor market, the more their career development and wage could be adversely affected. This could lead to increased difficulties in returning to jobs, declined opportunities for occupational moves, and motherhood wage penalties [54,55,56]. Maternal childcare is an important and significant barrier to off-farm employment of married Chinese women; public services for childcare can help alleviate this barrier [57]. Consequently, the traditional mother-dominated childcare model of infants and toddlers is unsustainable.

Secondly, less grandparents’ and fathers’ childcare (LGFC) has challenged the current family childcare arrangement. For a long time, even with a lack of formal childcare from nurseries, Chinese women’s participation in the labor force remained high because grandparents have carried a large share of the childrearing responsibilities by providing alternative no-charge services [58]. However, there are a few latent risks in grandparenting, mainly: migration and smaller family sizes have reduced the proportion of grandparents, adult children, and grandchildren living together, which could increase the difficulties of intergenerational care [59]; with delayed retirement and the aging population, grandparents are older and in poorer health when the second or third child is born, which will reduce the available time and their capacity for extra rearing [2,60]; excessive and longstanding grandchild care might also be harmful to the mental health and quality of life of older adults, leading to them withdrawing childcare provision [61,62]. Furthermore, there is a significant gender imbalance in early childhood care for generally absent paternal caregivers [18]. One study indicates that the parenthood wage penalty is higher for Chinese females than males, contributing to the gender wage gap [63]. Due to the weakening trend of grandparental and paternal childcare, Chinese women’s arrangements for childbirth, childrearing, and employment are more heavily constrained.

Finally, higher expectations of childcare quality (HECQ) are universal among young parents. Research has found that high-quality and scientifically based early education and care positively affect children’s growth and development, including school-related learning skills, physical health, cognitive ability, and other social skills [64,65,66]. Many grandparents often act as both caregivers and educators for grandchildren during preschool years, but the grandparental parenting style sometimes collides with that of their young adult children. Specifically, grandparents tend to focus on the basic needs of their grandchildren, such as eating, sleeping, dressing, bathing, and safety. In contrast, parents emphasize future achievements, such as discipline, personality, behavior, and academic performance [67]. Influenced by cultural origins and economic level, Chinese parents tend to have higher expectations for early childhood education and academic achievement than those in other countries, instead of paying much attention to daily care [68,69]. The most common reasons for Chinese parents sending young children to center-based nurseries are: primarily providing cultural or language learning (44.2%), followed by preparing children for school (42.0%), and then, providing care for working or schooling parents (39.7%), according to a survey [51]. Therefore, even in cases where family members can care for young children, parents have incentives to seek scientific and formal education and care services motivated by the need to promote high-quality growth and development of their children.

### 2.4. Present Study: Framework and Questions

Reviewing prior literature, although research has analyzed the characteristics and influencing factors of demand for childcare services, there are still several limitations or gaps. (1) Firstly, regional differences are a critical factor in the flexible implementation of public policies on childcare. There are few studies on the regional differences of demand for childcare services in China, especially the regional heterogeneity between urban and rural areas or eastern/central/western regions. (2) Secondly, unique Chinese social or cultural contexts need to be adequately incorporated into relevant studies about demand for childcare services, for instance, their roles in demand for childcare services’ regional disparities. More specifically, the family childcare context (FCC), as one of many diverse contexts in China, might play a unique role (causal mechanisms) in the formation of regional differences in demand for childcare services, and requires further research.

**Figure 2:**
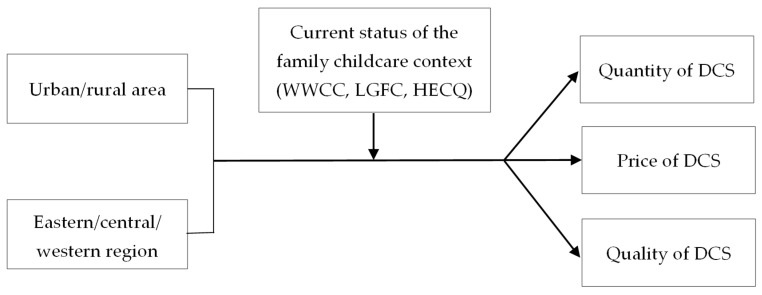
The moderating effect framework of FCC between RD and DCS.

**Figure 3:**
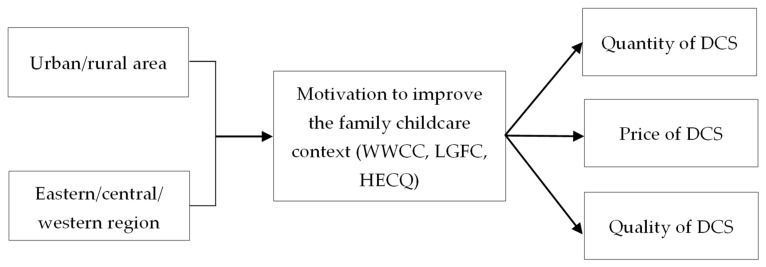
The mediating effect framework of FCC between RD and DCS.

Therefore, this research aims to explore the relationship between regional differences (urban/rural area, eastern/central/western region) and Chinese women’s DCS (quantity, price, quality) in addition to the causal mechanisms moderated and mediated by the family childcare context (WWCC, LGFC, HECQ) in mainland China. We designed two steps to explain the causal mechanisms: the first step is to examine the moderating effect of the “current status of the family childcare context” between RD and DCS (Figure 2). Then, the second step is to investigate the mediating effect of the “motivation to improve the family childcare context” between regional differences and demand for childcare services (Figure 3). Specifically, the following three research questions will guide our study.

1. What are the regional differences (urban/rural area, eastern/central/western region) in Chinese women’s demand for childcare services (quantity, price, quality)?
2. How does the current status of the family childcare context (WWCC, LGFC, HECQ) moderate the association between regional differences and demand for childcare services?
3. How does the motivation to improve the family childcare context (WWCC, LGFC, HECQ) mediate the association between regional differences and demand for childcare services?

## 3. Materials and Methods

### 3.1. Participants

This study uses secondary data from the National Population and Household Dynamics Monitoring Survey (NPHDMS) in 2019, a representative and authoritative survey organized by the National Health Commission of the People’s Republic of China. It implemented the probability proportional-to-size sampling (PPS) to recruit female participants aged 15–49. This article selected three provinces (Shanghai, Jiangsu, and Zhejiang) to represent eastern China, six provinces (Anhui, Shanxi, Jiangxi, Henan, Hubei, and Hunan) to represent central China, and four provinces (Chongqing, Yunnan, Sichuan, and Guizhou) to represent western China. The data from NPHDMS include basic demographic characteristics, childrearing situation, reproductive health and services, fertility willingness and family support, and other relevant information. Therefore, we were able to use these data to conduct our research. The participants of this study are women with children aged 0–3 years who have not enrolled in childcare centers (nurseries or kindergartens). This study does not cover women with children aged 0–3 who have enrolled in childcare centers and children over 3 years of age. After removing missing values and outliers, there were 1770 participants left for empirical analysis.

### 3.2. Measures

The dependent variable encompassed demand for childcare services (DCS), indicating the multidimensional needs of women with infants or toddlers from birth to age 3, measured along three dimensions: (a) quantity (“Do you plan to send your child under 3 to a nursery?” 0 = no, 1 = yes); (b) price (“How much can you afford for a nursery each month?” 0 = 0–1000 CNY, 1 = over 1000 CNY); (c) quality (“What quality factors do you care most about when you choose a nursery?” 0 = teacher capacity (education-related), 1 = safety conditions, level of nurturing, environment, sanitary conditions, or food nutrition (care-related)). It is worth mentioning that, for measuring the quality of DCS, most of the previous literature measured the quality of childcare services from structural and process quality perspectives [2]. However, according to the Chinese context, our study focused on participants’ preferences for education-related or care-related quality values.

The independent variables were urban/rural area and eastern/central/western region. Urban/rural area is a dummy variable, categorized by the participant’s type of household registration (0 = rural area = agricultural; 1 = urban area = non-agricultural). Agricultural registered participants usually live in rural areas (villages), and non-agricultural registered participants generally live in urban areas (cities, towns). Eastern/central/western region is also a dummy variable measured by the province in which the participant lives. The eastern area is the reference group, and the central and western areas are the control groups.

The moderating variable focused on the current status of the family childcare context (FCC). To explore the moderating effect of FCC between RD and DCS, this research evaluated the current FCC from three perspectives: (a) the current WWCC, measured by maternity leave days (“How many days were you actually on maternity leave when giving birth to the child?” 0 = 0–200 days, 1 = over 200 days) and job adjustment (“Has your job been adjusted due to birth of the child?” 0 = no, 1 = yes); (b) the current LGFC, measured by senior grandparents’ care (“Who was the primary caregiver of the child during the day last month?” 0 = others, 1 = senior grandparents) and paternal company time (“How many hours did the father accompany the child last week?” 0 = 0–20 h, 1 = over 20 h); (c) the current status of HECQ, measured by the entry of early learning classes (“Has the child entered any early learning classes?” 0 = no, 1 = yes).

The mediating variable was the motivation to improve the family childcare context (FCC). To explore the mediating role of the FCC on the relationship, mediating variables were measured by women’s reasons or motivations to improve the FCC. Based on the participants’ replies to the question “What are the main reasons why you want to send your child to a nursery?”, this paper divides the reasons into three types: (a) the motivation to improve WWCC (no time to care for the child, affects career development, too tired to care for the child); (b) the motivation to improve LGFC (grandparents are unwilling to care for the child, grandparents are unable to care for the child); (c) the motivation to improve HECQ (to provide the child with professional care, to provide the child with peers).

For controlled variables, under the current research context, we predict that other variables would affect the outcomes. Therefore, to ensure the accuracy of the conclusions, this study controlled for the following variables: (a) the mother’s characteristics (education, age, age squared, ethnicity, job, marital status, migrant, log of individual income); (b) the child’s characteristics (gender, age, singleton); (c) the family’s characteristics (log of household income per capita, mother as the primary caregiver or not, living with grandparents or not). Table 1 displays the descriptive statistics of the main variables, with detailed variable definitions and coding.

### 3.3. Data Analysis

STATA software (version 16) was used for all data analyses. First, we conducted a descriptive analysis of the basic characteristics of childcare arrangements and demand in Chinese families. Secondly, this study applied logit regression to analyze benchmark and moderating effects. According to the above measurements, the independent variable (quantity, price, or quality of DCS) is binary. The logit regression model focuses on the odds ratio (OR) of events rather than probability. For example, assuming p is the probability of “plan to send the child to a nursery before age 3”, 1 − p is the opposite, and the OR is p/(1 − p). To examine the association between regional differences and demand for childcare services, this paper builds the following regression equation:

log[p_i_/(1 − p_i_)] = DCS_i_ = α_0_ + α_1_URA_i_ + α_2_ECWA_i_ + β_i_X_i_ + ε_i_ (1)

Specifically, i represents the individual participant. p_i_ is the probability of DCS_i_ being equal to 1. DCS_i_ denotes the demand for childcare services of the participant. URA_i_ is the household registration type (urban/rural area) of the participant. ECWA_i_ denotes the geographical region (eastern/central/western) where the participant lives. X_i_ denotes the matrix of control variables that affect the participant’s outcomes. α_0_ represents the constant term of the model; α_1_, α_2_, and β_i_ are the coefficients of corresponding variables; and ε_i_ denotes the residual term.

Third, to further explore how regional differences affect demand for childcare services against mediating variables, we adopted the multiple mediating effect model referenced in prior studies [70,71]. The regression models are as follows:

Y_i_ = α_0_ + α_1_D_i_ + α_2_X_i_ + ε_i_ (2)

M_i_ = β_0_ + β_1_D_i_ + β_2_X_i_ + ε_i_ (3)

Y_i_ = γ_0_ + γ_1_D_i_ + γ_2_M_i_ + γ_3_X_i_ + ε_i_ (4)

Y_i_ = (γ_0_ + γ_2_β_0_) + (γ_1_ + γ_2_β_1_)D_i_ + (γ_3_ + γ_2_β_2_)X_i_ + ε_i_ (5)

Among them, Equation (2) is the benchmark model; Y_i_ denotes the dependent variable (DCS), and D_i_ represents the independent variable (RD). Equations (3)–(5) are the mediating models. M_i_ is the mediating variable (motivation to improve WWCC, LGFC, and HECQ); β_1_ in Equation (3) denotes the direct impact of independent variable D_i_ on mediating variable M_i_; γ_1_ in Equation (4) denotes the direct effect of the independent variable D_i_ on the dependent variable Y_i_; γ_2_ in Equation (4) denotes the direct effect of mediating variable M_i_ on dependent variable Y_i_. Finally, γ_2_β_1_ in Equation (5) indicates the indirect effect of the independent variable D_i_ on the dependent variable Y_i_.

To demonstrate the mediation effects, four conditions should be met. First, in Equation (2), there should be an overall treatment effect demonstrated on the dependent variable, that is, α_1_ is significant. Second, in Equation (3), there should be a treatment effect on the mediator, that is, β_1_ is significant. Third, in Equation (4), there should be an effect of the mediator on the dependent variable controlling for the treatment, that is, γ_2_ is significant. Fourth, in Equation (4), the residual direct effect of the treatment variable on the dependent variable (γ_1_) should be smaller (absolute value) than the overall treatment effect in Equation (2) (α_1_) [70].

## 4. Results

### 4.1. The Characteristics of Childcare Arrangements and Demands

Regarding current childcare arrangements, only 4.1% of Chinese children aged birth to 3 years of age are enrolled in center-based nurseries, reported by their mothers. The urban enrollment rate (5.3%) is higher than the rural one (2.7%), while there is a minimal difference between eastern (4.4%), central (4.0%), and western (4.2%) China. Among infants and toddlers who do not attend nurseries, 63.9% of them are primarily cared for by their mothers, 26.4% by their paternal grandparents, 7.2% by their maternal grandparents, 1.4% by their fathers, and 1.1% by others. The reasons for failure to find an appropriate nursery include disagreement from family members (45.9%), being too young to be accepted (30.8%), expensive fees (15.7%), far distance or lack of nursery nearby (12.9%), concern about quality (12.6%) and others (17.3%). As for the attributes of childcare facilities, approximately 90% of them are privately funded, charging over 1500 CNY monthly; the rest are publicly funded, charging below 1000 CNY monthly. Concerning location, more than 85% of enrolled children are in nurseries near their community, and a small proportion are near their parents’ workplaces.

For demand for care services, 24.0% of mothers of children under three show willingness to place their child in childcare centers before age 3, which is 20% higher than the actual enrollment rate. Nurseries are needed most for toddlers between 2 and 3 years (22.1%), compared with between 0 and 2 years (1.9%). Women in urban areas (27.1%) have a higher need to send their children to nurseries before preschool than in rural areas (20.5%). In eastern China (32.2%), women act more urgently in placing their children in nurseries than in central (23.6%) and western (21.1%) China. In this study, 38.0% of participants reported an affordable service price of 500 to 1000 CNY, followed by 0 to 500 CNY (35.8%), 1000 to 2000 CNY (22.8%), and over 2000 CNY (3.4%). The proportion of urban women (36.3%) who can afford a price of above 1000 CNY is clearly higher than the proportion of women in rural areas (15.3%). Similarly, mothers in eastern China appear to pay a higher cost for services than in central and western China. In addition, when women find it challenging to choose a nursery, safety conditions (41.7%) are the most potent determinant of quality. Other quality factors include teacher capacity (28.5%), environment (12.0%), nurturing level (10.0%), sanitary conditions (4.7%), and food nutrition (3.1%). However, the characteristics of quality demand are relatively homogeneous and do not show significant disparities between regions.

### 4.2. Benchmark Regression

Table 2 displays the results of the benchmark regression analysis for demand for childcare services from three perspectives: quantity, price, and quality. In Model 1, urban women have higher quantity needs for DCS than rural women, but this result is not statistically significant when controlling all variables. Women in eastern areas are 38.1% (β = −0.481, OR = 1.381, *p* < 0.001) more likely than those in central areas and 40.4% (β = −0.518, OR = 1.404, *p* < 0.001) more likely than those in western areas to send their children to nurseries before three years of age. As for price, in Model 2, urban women are 54.3% (β = 0.434, OR = 1.543, *p* < 0.001)) more likely than rural women to pay a price of over 1000 CNY each month for childcare. However, the price aspect of DCS appears to be similar among eastern, central, and western China. Model 3 reveals that there is no statistically significant difference in quality demand for childcare services between urban and rural areas (*p* > 0.1) and eastern, central, and western regions (*p* > 0.1). In other words, no solid evidence proves that women’s preference for care-related or education-related quality varies among regions. In addition, participants of a higher education level, older age, Han ethnicity, non-public department employee, unmarried, younger child age, non-only child, and mother as the primary caregiver show stronger needs for non-family childcare.

### 4.3. The Moderating Role of FCC

To examine the influencing mechanism between regional differences and demand for childcare services, this study separated the samples based on the current status of WWCC, LGFC, and HECQ. Then, we analyzed their moderating roles between subsamples. As shown in Table 3, in Models 4–5, the reduction in female maternity leave days significantly widens the gaps in the quantity of DCS between eastern, central, and western regions (for central, from β = −0.223 to β = −0.730; for western, from β = −0.359 to β = −0.650). In Models 6–7, job adjustment due to childbirth also significantly widens the gaps in the quantity of DCS between eastern, central, and western regions (for central, from β = −0.433 to β = −1.005; for western, from β = −0.536 to β = −0.774). There is no significant difference in quantity between urban and rural areas. Models 8–9 report that the urban–rural gap in affordability becomes more prominent as the days of maternity leave decline (from β = 0.339 to β = 0.579). Job adjustment can also bring about the same effect (from β = 0.437 to β = 0.719) (Models 10–11). However, the differences in the price of DCS are not significant between eastern, central, and western China (*p* > 0.1). Overall, maternity leave days and job adjustment have solid moderating effects on the regional differences in DCS.

As shown in Table 4, childcare from other family members (grandparents and fathers) is a crucial contributor to disparities among women’s demand for childcare services (DCS). Models 12–13 reveal that the weakening of grandparental childcare provision increases the gap in quantity of DCS between eastern, central, and western China (for central, from β = −0.269 to β = −0.623; for western, from β = −0.491 to β = −0.517). Similarly, urban women with less grandparental help parenting the child have to accept higher childcare prices (from β = 0.274 to β = 0.695) (Models 16–17). Models 14–15 indicate that regional differences in the quantity of DCS rise as fatherly company hours decrease, both between urban and rural areas (from β = −0.062 to β = 0.345) and the eastern, central, and western regions (for central, from β = −0.372 to β = −0.580; for western, from β = −0.580 to β = −0.593). In Models 18–19, the urban–rural difference in willingness to pay more for childcare in the sample with fewer paternal company hours is slightly larger than in the sample with more fatherhood care hours (from β = 0.410 to β = 0.499). Grandparental and paternal caregiving both play moderating roles between RD and DCS.

Table 5 displays the impact of early education-related factors on female demand for childcare programs. This section analyses the moderating effect of entering or not entering early learning classes between regional differences and childcare demand. For mothers whose children did not enroll in early learning classes, differences between the eastern, central, and western regions still exist in the quantity of DCS (for central, β = −0.446; for western, β = −0.532) (Model 20). Urban–rural differences in the price of DCS remain significant (β = 0.388) (Model 22), and RD have no significant impact on the quality of DCS in the participants (*p* > 0.1) (Model 24). However, in the sample that entered early education classes, there are no significant regional differences in the quantity, price, and quality of DCS (*p* > 0.1) (Models 21, 23, and 25). The main reason for such phenomena might be that early learning classes are a solid alternative to childcare services that have met women’s demands. In practice, some educated or wealthy parents do not place their toddlers in center-based nurseries but in private early learning classes to develop their cognitive, language, and learning skills.

### 4.4. The Mediating Role of FCC

**Figure 4:**
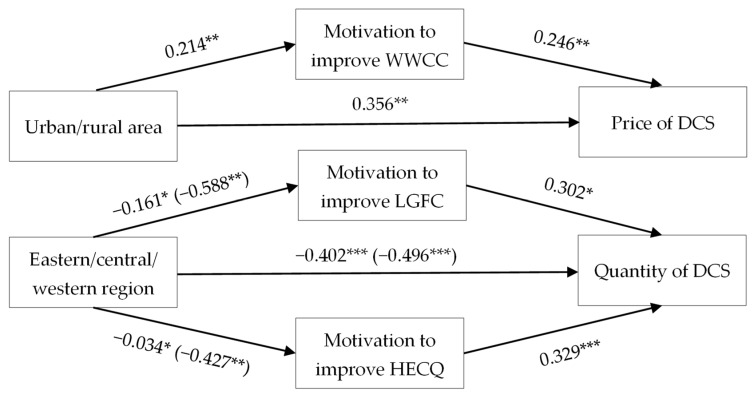
The mediating roadmap of FCC between RD and DCS. Note: * *p* < 0.1, ** *p* < 0.05, *** *p* < 0.01. The item inside parenthesis belongs to the western region, and the outside one belongs to the central region.

This study conducted a mediation analysis of the motivation to improve the FCC in order to further explore the intermediate mechanism of how regional differences affects demand for childcare services (Table 6). Model 26 reports that urban women have stronger motivation to improve WWCC than rural women (β = 0.214, OR = 1.239, *p* = 0.032). Meanwhile, urban and rural areas (β = 0.356, OR = 1.428, *p* < 0.001) and motivation to improve WWCC (β = 0.246, OR = 1.279, *p* = 0.018) significantly affect the price of DCS (Model 30). The four mediating conditions were all met, showing a significant mediating effect of the motivation to improve WWCC on the relationship between RD and the price of DCS. The direct effect of urban/rural area on the price of demand is 0.356, and the indirect effect through the mediator “motivation to improve WWCC” is 0.214 × 0.246 (Figure 4). In brief, urban women have stronger motivation to improve career–child conflicts and are willing to pay a higher cost for alternatives.

Similarly, Models 27–28 reveal that women in the eastern region are more likely to improve LGFC and HECQ than those in the central and western regions. Model 29 shows that eastern/central/western area, motivation to improve LGFC, and motivation to improve HECQ all statistically significantly impact the quantity of DCS. It met all four conditions of mediation, meaning the motivation to improve LGFC and HECQ plays mediating roles between eastern/central/western region and the quantity of DCS. The direct effect of eastern/central/western region on the quantity of DCS is −0.402 (−0.496); the indirect effect through the mediating motivation to improve LGFC is −0.161 × 0.302 (−0.588 × 0.302), and motivation to improve HECQ is −0.034 × 0.329 (−0.427 × 0.329) (Figure 4). Females in eastern China have stronger motivation to improve LGFC and HECQ; therefore, they have more substantial needs for social services.

## 5. Discussion and Implications

Childcare services continue to be a thorny issue in mainland China after being neglected for several decades, despite serving as a critical fertility and family support measure for women with children before preschool. In the new era of the Three Child Policy and the aging population, it is necessary to gain a better understanding of the demand features and boost the high-quality development of childcare services. Based on a national quantitative research design, our study has found that the quantity of childcare demand differs significantly among geographical regions, and the price of demand varies considerably between urban and rural areas. Furthermore, moderation and mediation analyses indicated that these discrepancies could result from family childcare settings. This section discusses the main findings and possible implications for policy and practice.

### 5.1. Maternal Demand Preferences for Childcare Services

The childcare needs of Chinese mothers are more pressing than those of fathers and deserve more attention, under the traditional family division pattern of “men’s work centers around outside, and women’s work centers around the home”. Findings on demand characteristics for childcare from this research support previous claims. In 2019, less than 5 percent of children from birth to three years enrolled in center-based nurseries in China; the demand is vastly higher than the actual enrollment figures [18]. Mothers with babies face challenges in finding easy-access, low-cost, and high-quality institutions, with the primary caregivers being the mothers and grandparents during the day [51]. Privately funded nurseries currently represent the majority of childcare institutions in China, while publicly funded nurseries charge the lowest average fees. People generally show a high level of trust in public nurseries but lack confidence in private institutions [11]. The demand for full-day care close to home is the most sought after, which is convenient for working parents or grandparents to pick up their children [11,51]. Women are most concerned about safety conditions, distance from home, and teacher capacity, showing a high requirement for service quality [36]. Furthermore, most mothers with toddlers are reluctant to place their children in nurseries before they are two years old, which is the best starting age. Separating children from their mothers at a very young age is disadvantageous for their physical and mental health; at the same time, childcare programs at younger ages are more expensive for consumers.

Additionally, some demographic variables predict maternal demands for public childcare, which is consistent with many studies. For the quantity of DCS, females with a higher education level, older age, working in a private company, unmarried, non-only child, and being the primary caregiver are more eager to use non-family childcare [38]. For the price of DCS, higher education, older age, only child, and higher individual and household income indicate a higher likelihood for mothers to afford the high monetary costs of childcare programs. It is worth mentioning that participants with one child have a higher need for care services but a lower capacity to pay for them. This indicates that under the new fertility policy in the future, the demand for care services and the payment ability for the second or third child are contradictory, which implies that more public support is needed [16]. For the quality of DCS, mothers of higher education, younger age, Han ethnicity, married, and from a wealthy family are more likely to emphasize quality related to education, in line with prior literature [36]. However, some relationships above are different from previous research. For instance, Hong et al. [51] found that parental age, child age, and singleton birth did not statistically significantly predict the likelihood of non-parental care, requiring further correction and explanation in follow-up research. The above findings will facilitate a more targeted approach to meeting the childcare needs of specific groups of women.

### 5.2. Regional Differences in DCS and Prioritized Strategy

As the first comprehensive study on regional differences in Chinese childcare needs, our study has found a few meaningful results. Firstly, our finding does not align with the current literature that urban parents show more urgent needs for childcare services than rural parents [11,12]. This study shows that the quantity of DCS does not differ statistically significantly between urban and rural regions. However, given that other relevant variables are held constant, urban women are willing to pay higher childcare prices for infants and toddlers than rural women. This association may explain why the actual urban enrolment rate is higher than the rural enrolment rate: when the quantity of demand remains the same, rural women have lower payment capacity than urban women, which leads to lower effective demand for services. Secondly, women in eastern China (considered a more developed region) are more willing to send their children to center-based programs than women in central/western China (less developed). This shows new relationships that previous studies have not found and fills research gaps. Thirdly, there is no significant difference between urban and rural areas or among eastern, central and western regions in terms of women’s preference for care-related or education-related quality, which provides new evidence for the importance of ensuring the quality of childcare across all areas [21].

These findings shed new light on promoting the sustainable development of childcare services in China. Firstly, we should broaden funding sources for childcare programs. More public funds are needed to expand the proportion of public institutions or kindergartens should be encouraged to run welfare childcare services for under 3 s. At the same time, private capital is also encouraged to expand private facilities, which could provide various services and improve the quality of established facilities. The central and provincial governments should inject more fiscal budget into areas with poor financial conditions, building a reasonable and sustainable fiscal system at all levels. One study also shows that in early childhood education, “puhui” kindergartens with satisfactory quality but lack of equality are not an appropriate solution in China, calling for equal treatment and corresponding proportional funds [72]. Secondly, local governments should adopt a differentiated regional strategy, promoting childcare services in sequence: “availability prioritized” or “affordability prioritized”. Availability of quantity and affordability of price are two sides of the childcare services coin, which are difficult to satisfy simultaneously [2]. Implementing a strategy of prioritizing affordability or accessibility in certain regions might be a feasible solution to the “3A” problems. More specifically, in areas with limited funds, such as rural and central and western China, the strategy of “affordability prioritized” should be adopted. The price of childcare programs should first be reduced so that a small number of families with urgent needs can enjoy the benefits, which will help stimulate the popularity of new care arrangements. Next, the quantity of affordable childcare should gradually increase to expand the benefits to a wider population. On the other hand, urban and eastern China should adopt the strategy of “availability prioritized”. They should first provide sufficient accessible childcare programs to the many families in urgent need. Then, they should reduce service fees step by step by injecting public funding. In the future, China should gradually extend provision from the eastern and urban areas to the central and western and rural areas, setting up a diversified supply system of childcare services.

### 5.3. The Role of Family Childcare Context and Targeted Services

There are various reasons to consider using formal childcare in China: parental employment, child development, fertility choices, elderly health, generational relations, etc. As a matter of fact, the family childcare arrangements are involved in a complex and dynamic context. Our findings add a new perspective on the complicated role of family childcare settings between regional differences and demand for infant/toddler care. In line with prior studies [73,74], the moderating effect analysis shows that the current status of family childcare settings could impact the regional differences in women’s needs for childcare programs. Furthermore, exploring the mediating effect reveals that urban women are more willing to pay for childcare than rural women because of stronger motivation to improve WWCC. However, Chinese female employment rights and interests are not well-protected, as childbirth and childrearing often lead to adverse career outcomes, such as unemployment, low wages, and blocked career advancement [53]. Urban women’s labor force participation and the opportunity cost of childcare are significantly higher than those of rural women, so they are more eager to pay for care programs rather than give up their jobs [56,57]. As an essential element of family-friendly policies, childcare services are a fundamental approach to alleviating work–child conflicts for women. Therefore, these urban working mothers who face issues with an unfavorable work–child balance might resort to more support from social services when raising a child.

Moreover, the analyses also show that mothers in eastern China need to send their very young children to nurseries more than mothers in central and western China. Women in eastern China have a stronger motivation to improve LGFC (grandparents are unwilling or unable to care for the child) and HECQ (to provide the child with professional care and peers). Considering the aging population, delayed retirement, and migration, it is increasingly common for grandparents to be unwilling or unable to care for their grandchildren [62]. Even though the prevalence of grandparental childcare has decreased, it is still one irreplaceable childcare choice in the Chinese context [75,76]. There is gender inequality in terms of housework and childcare with the universal absence of fatherhood, which is more prominent in economically developed areas such as eastern China [10]. In addition, educated females (widely distributed in developed areas) are more likely to pay for childcare programs, have a more open mind about parenting, and have a higher degree of acceptance of the emerging care arrangement [38]. Therefore, women’s needs for childcare programs in eastern China are more urgent due to the lack of childcare support and the positive pursuit of high-quality childrearing.

To accommodate family childcare situations, the layout, pattern, and quality of nurseries need to be improved in a targeted way. Firstly, regarding the layout of facilities, priority should be given to planning childcare centers near communities or workplaces. Community-based childcare facilities need to be adopted, expanding the student pool for institutions and reducing the time costs of drop-off and pick-up for families. The government can support qualified large- and medium-sized enterprises to cooperate with communities to jointly operate childcare centers, aiming to reduce the burden on dual-income families. At the same time, governments could finance enterprises to equip mother and baby facilities and create a childbirth-friendly corporate culture. Secondly, centers should provide programs with flexible patterns. Services with flexible durations such as full-day, half-day, and temporary care are needed. Elderly grandparents are tired from intensive intergenerational parenting, and this flexibility will help relieve their physical and mental exhaustion. Planners could incorporate childcare into senior care to reduce the operating costs of institutions and the care burden of working females for young children and the elderly. Concerning quality, administrators should integrate the custodial and educational functions of childcare services and promote the integrated development of care and education. In response to inadequate work experience and capacity of staff and teachers, qualification systems, standards, ratings, training, and supervision should be established. The nurseries should follow the progression of child development and set curriculums in a targeted and scientific manner to facilitate the overall development of infants and toddlers.

## 6. Conclusions and Limitations

To conclude, based on the data from NPHDMS, this paper analyzes the regional differences in the quantity, price, and quality of demand for childcare services of infants and toddlers aged 0 to 3 among Chinese women, involving the family childcare context of stakeholders, and draws the following conclusions. First, urban females are willing to pay higher service fees for center-based nurseries than rural females due to the more salient work–childcare conflict faced by urban mothers. Growing work–child conflicts appear to increase women’s need for childcare services and their willingness to pay higher fees. Second, females in eastern China have a stronger need to send their children to center-based nurseries before the age of three compared to females in central and western China, as a result of a lack of grandparental and paternal care and the expectation of higher quality childcare faced by mothers in eastern China. The difficulty for grandparents to participate in the care of young children and the absence of fatherly responsibility encourage women to seek public services. However, there is no notable regional difference in terms of maternal preference for care-related or education-related quality. Overall, significant regional discrepancies exist in Chinese childbearing females’ demand for childcare services from birth to 3 years, to some extent, caused by the childcare circumstance of different families. To resolve the current dilemma of childcare services, the regional differences should be fully considered, and the development of childcare services could be promoted by implementing regional prioritization strategies and targeted services. Nonetheless, the above countermeasure is a compromise to ease the childcare supply and demand conflict, which needs to be adapted to local conditions.

There are several limitations in our research that are worth mentioning. The first limitation relates to the participants due to our data sources. This article only focuses on the need for childcare services from the perspective of women. However, women’s needs are always affected by partners, grandparents, neighbors, or other stakeholders, and decisions on formal or informal childcare arrangements are often made by them together. The second limitation involves the measurement of the dependent variables. In this study, demands for childcare services are divided into three dimensions: quantity, price, and quality. There may be a few demand characteristics that need to be appropriately included such as preferences for the nursery’s location and operation time. In addition, this study measures quality only from the perspective of preference for care-related or education-related aspects based on available materials. The third limitation concerns using cross-sectional data instead of longitudinal data in our research. Future studies should use longitudinal methods to collect relevant data and demonstrate changes in the demand for childcare services over time. Lastly, the COVID-19 pandemic’s immense negative impact on the service sectors has not been included. In particular, China’s strict epidemic prevention and control policy has resulted in thousands of privately owned childcare institutions struggling to survive or even closing down. Nevertheless, this study contributes new recommendations for resolving the “3A” problems of childcare services for policymaking and practice in Chinese societies.

## Figures and Tables

**Table 1:** The main variable measures and descriptive statistics (N = 1770).

Variable			Definition and Coding	Percent/Mean
Dependent variable	Quantity of DCS		Plan to send the child to a nursery before age 3 = 1, Otherwise = 0	23.99%
	Price of DCS		Affordable price of a nursery > 1000 CNY = 1, Otherwise = 0	26.24%
	Quality of DCS		Emphasis on care-related quality = 1, Emphasis on education-related quality = 0	72.20%
Independent variable	Urban/rural area		Urban area = 1, Rural area = 0	53.08%
	Eastern region		Reference group	13.37%
	Central region		Control group	53.11%
	Western region		Control group	33.52%
Moderating variable	Current status of WWCC		Maternity leave > 200 days = 1, Otherwise = 0; Job adjustment = 1, Otherwise = 0	41.82%; 4.74%
	Current status of LGFC		Senior grandparents as primary caregivers during the day = 1, Otherwise = 0; Fatherly accompany time > 20 h = 1, Otherwise = 0	45.16%; 49.18%
	Current status of HECQ		Entry of early learning classes = 1, Otherwise = 0	4.21%
Mediating variable	Motivation to improve WWCC		Yes = 1, No = 0	31.09%
	Motivation to improve LGFC		Yes = 1, No = 0	8.10%
	Motivation to improve HECQ		Yes = 1, No = 0	54.38%
Controlled variable (mother)	Education level		1 (lowest)–8 (highest)	4.00
	Age		Participant age (years) in 2019	30.12
	Age squared		To fit the nonlinear equation	932.22
	Race		Han = 1, Otherwise = 0	92.63%
	Job	Public department	Reference group	24.73%
		Public enterprise	Control group	8.24%
		Private enterprise	Control group	24.23%
		Self-employed	Control group	42.79%
	Marital status		Married = 1, Otherwise = 0	99.47%
	Migrant		Migrant population = 1, Otherwise = 0	29.33%
	Log of individual income		To correct the distribution of the variable	5.83
Controlled variable (child)	Child gender		Boys = 1, Girls = 0	52.10%
	Child age		Child age (years) in 2019	1.58
	Singleton		Non-only child = 1, Only child = 0	51.51%
Controlled variable (family)	Log of household income per capita		To correct the distribution of the variable	9.62
	Primary caregiver		Mother as primary caregiver = 1, Otherwise = 0	60.96%
	Living arrangement		Living with grandparents = 1, Otherwise = 0	56.09%

Note: The percentage describes the grouping characteristics when the coding is 1.

**Table 2:** The results of benchmark regression.

Variables	Quantity of DCS	Price of DCS	Quality of DCS
	Model 1	Model 2	Model 3
Urban/rural area	0.141 (0.133)	0.434 *** (0.155)	0.052 (0.134)
Eastern region (Reference group)			
Central region	−0.481 *** (0.157)	−0.228 (0.190)	−0.170 (0.161)
Western region	−0.518 *** (0.169)	−0.230 (0.200)	−0.064 (0.177)
Education level	0.178 *** (0.060)	0.322 *** (0.071)	−0.110 * (0.063)
Age	0.188 * (0.112)	0.274 * (0.150)	0.209 * (0.112)
Age squared	−0.003 (0.002)	−0.004 * (0.002)	−0.003 * (0.002)
Race	0.365 * (0.213)	−0.392 (0.246)	−0.482 ** (0.237)
Public department (Reference group)			
Public enterprise	0.349 * (0.201)	0.180 (0.228)	0.026 (0.209)
Private enterprise	0.297 * (0.158)	0.285 (0.183)	−0.032 (0.161)
Self-employed	0.293 * (0.177)	0.249 (0.198)	0.210 (0.180)
Marital status	−1.314 ** (0.568)	−0.710 (0.732)	1.136 ** (0.549)
Migrant	0.061 (0.112)	0.213 (0.132)	0.051 (0.118)
Log of individual income	0.003 (0.027)	0.085 ** (0.036)	−0.008 (0.029)
Child gender	0.012 (0.102)	0.146 (0.125)	0.210 * (0.108)
Child age	−0.425 *** (0.064)	−0.208 *** (0.079)	−0.114 (0.071)
Singleton	0.334 *** (0.110)	−0.239 * (0.136)	−0.065 (0.121)
Log of household income per capita	−0.010 (0.090)	0.998 *** (0.126)	−0.217 ** (0.091)
Primary caregiver	0.282 ** (0.133)	−0.256 (0.160)	−0.062 (0.140)
Living arrangement	0.041 (0.108)	0.155 (0.129)	0.018 (0.112)
Constant	−2.285 (1.955)	−14.391 *** (2.724)	−0.277 (1.926)
Adj_R^2^	0.063	0.200	0.028
Observations	1770	1613	1752

Note: * *p* < 0.1, ** *p* < 0.05, *** *p* < 0.01. The number in parenthesis is the standard error.

**Table 3:** The moderating effect of current status of WWCC.

Variables	Quantity of DCS				Price of DCS			
	Model 4	Model 5	Model 6	Model 7	Model 8	Model 9	Model 10	Model 11
	Maternity Leave		Job Adjustment		Maternity Leave		Job Adjustment	
	>200 d	≤200 d	No	Yes	>200 d	≤200 d	No	Yes
Urban/rural area	0.018 (0.173)	0.311 (0.221)	0.116 (0.140)	0.697 (0.529)	0.339 (0.218)	0.579 ** (0.237)	0.437 ** (0.164)	0.719 * (0.629)
Eastern region (Reference group)								
Central region	−0.223 (0.261)	−0.730 *** (0.208)	−0.433 *** (0.168)	−1.005 ** (0.509)	0.206 (0.336)	−0.470 (0.244)	−0.186 (0.202)	−0.834 (0.690)
Western region	−0.359 (0.278)	−0.650 *** (0.224)	−0.536 *** (0.181)	−0.774 * (0.600)	−0.272 (0.352)	−0.076 (0.264)	−0.272 (0.212)	−0.019 (0.706)
Controlled variables (Yes)								
Adj_R^2^	0.046	0.075	0.059	0.168	0.175	0.142	0.195	0.297
Observations	938	832	1591	179	881	732	1457	156

Note: * *p* < 0.1, ** *p* < 0.05, *** *p* < 0.01. The number in parenthesis is the standard error.

**Table 4:** The moderating effect of the current status of LGFC.

Variables	Quantity of DCS				Price of DCS			
	Model 12	Model 13	Model 14	Model 15	Model 16	Model 17	Model 18	Model 19
	Senior Caregivers		Fatherly Accompany		Senior Caregivers		Fatherly Accompany	
	Yes	No	>20 h	≤20 h	Yes	No	>20 h	≤20 h
Urban/rural area	0.327 (0.209)	−0.114 (0.202)	−0.062 (0.187)	0.345 * (0.191)	0.274 ** (0.243)	0.695 ** (0.233)	0.410 * (0.213)	0.499 ** (0.243)
Eastern region (Reference group)								
Central region	−0.269 (0.263)	−0.623 *** (0.230)	−0.372 * (0.212)	−0.580 ** (0.234)	−0.256 (0.312)	−0.371 (0.270)	−0.380 (0.256)	−0.029 (0.298)
Western region	−0.491 * (0.278)	−0.517 ** (0.248)	−0.580 ** (0.229)	−0.593 ** (0.251)	−0.089 (0.318)	−0.370 (0.288)	−0.352 (0.269)	−0.038 (0.313)
Controlled variables (Yes)								
Adj_R^2^	0.073	0.078	0.071	0.074	0.232	0.174	0.221	0.187
Observations	713	844	942	828	654	774	843	770

Note: * *p* < 0.1, ** *p* < 0.05, *** *p* < 0.01. The number in parenthesis is the standard error.

**Table 5:** The moderating effect of the current status of HECQ.

Variables	Quantity of DCS		Price of DCS		Quality of DCS	
	Model 20	Model 21	Model 22	Model 23	Model 24	Model 25
	Early Learning Classes		Early Learning Classes		Early Learning Classes	
	No	Yes	No	Yes	No	Yes
Urban/rural area	0.165 (0.136)	0.000 (0.687)	0.388 ** (0.158)	1.523 (1.119)	0.058 (0.138)	0.189 (0.719)
Eastern region (Reference group)						
Central region	−0.446 *** (0.166)	−0.750 (0.579)	−0.225 (0.200)	0.693 (0.923)	−0.144 (0.170)	−0.448 (0.610)
Western region	−0.532 *** (0.180)	−0.190 (0.613)	−0.237 (0.212)	0.719 (1.031)	−0.011 (0.188)	−1.004 (0.702)
Controlled variables (Yes)						
Adj_R^2^	0.062	0.156	0.183	0.379	0.028	0.110
Observations	1655	115	1522	189	1637	115

Note: ** *p* < 0.05, *** *p* < 0.01. The number in parenthesis is the standard error.

**Table 6:** The mediating effect of motivation to improve FCC.

Variables	Motivation to Improve WWCC	Motivation to Improve LGFC	Motivation to Improve HECQ	Quantity of DCS	Price of DCS	Quality of DCS
	Model 26	Model 27	Model 28	Model 29	Model 30	Model 31
Urban/rural area	0.214 ** (0.129)	−0.117 (0.199)	0.115 ** (0.125)	0.155 (0.134)	0.356 *** (0.156)	0.041 (0.134)
Eastern region (Reference group)						
Central region	0.252 (0.164)	−0.161 * (0.246)	−0.034 * (0.159)	−0.402 *** (0.157)	−0.208 (0.191)	−0.178 (0.162)
Western region	0.150 (0.176)	−0.588 ** (0.284)	−0.427 ** (0.168)	−0.496 *** (0.170)	−0.215 (0.202)	−0.084 (0.178)
Motivation to improve WWCC				0.323 *** (0.110)	0.246 ** (0.136)	0.051 (0.118)
Motivation to improve LGFC				0.302 * (0.176)	0.182 (0.223)	0.339 * (0.200)
Motivation to improve HECQ				0.329 *** (0.109)	0.040 (0.131)	−0.228 ** (0.114)
Controlled variables (Yes)						
Adj_R^2^	0.071	0.081	0.065	0.071	0.202	0.032
Observations	1770	1770	1770	1770	1613	1752

Note: * *p* < 0.1, ** *p* < 0.05, *** *p* < 0.01. The number in parenthesis is the standard error.

## Data Availability

The data used to support the findings of this study are available from the corresponding author upon request (e-mail: chuanfangchenhust@163.com).
